# Broad spectrum integration of climate change in health sciences curricula

**DOI:** 10.3389/fpubh.2022.954025

**Published:** 2022-07-25

**Authors:** Oladele A. Ogunseitan

**Affiliations:** Department of Population Health and Disease Prevention, University of California, Irvine, Irvine, CA, United States

**Keywords:** climate change, education, health sciences, curriculum integration, medicine, public health, nursing science, pharmacy and pharmaceutical sciences

## Abstract

In response to a University of California systemwide initiative to expand the knowledge base of climate change, two half-day workshops were held for faculty in the College of Health Sciences at the UC Irvine. In the first workshop, 20 participants who teach in the Schools of Nursing, Medicine, Pharmacy, and Pharmaceutical Science, or the Program in Public Health convened to explore concepts of sustainability, theoretical models of curriculum integration, challenges to adding new competencies into professional training, and strategies for integrating climate change modules and case studies into the curricula. The second half-day workshop was held a year after the first workshop to review how faculty members have modified their syllabus to integrate climate change information with varying degrees of success. A case study is presented regarding an asynchronous fully online course Introduction to Global Health, which is open to enrollment by students from all campuses of the University of California. The outcomes revealed preferential adoption of models of curriculum integration which minimized disruption of the sequence of topics in pre-existing courses. These include, for example, the use of longitudinal climate datasets for quantitative analysis of disease outcomes, and description of episodic events involving extreme weather conditions to explore differences in social determinants of vulnerability to climate change impacts in different populations. Integration of climate change as a distinct topic seems easier in elective courses in comparison with required courses designed to cover pre-established professional knowledge, competencies, and skills. The emergent requirement for interprofessional training in the health sciences provides an opportunity for the development of a cross-cutting competency domain including climate change as a unifying theme in a stand-alone course or set of courses in a sequenced model of curriculum integration.

## Introduction

The early framing of international response to climate change, for example through the reports of the Intergovernmental Panel on Climate Change, emphasized assessments to confirm the scientific basis of human contributions. In comparison to approaches to mitigation, issues related to impacts, adaptation, and vulnerability were even less understood in part due to highly variable characteristics of populations distributed globally and confounding with preexisting conditions (1). Specifically, the framing of the health impacts of climate change focused on the range of vector-borne diseases such as malaria, and on the exacerbation of pre-existing burden of such diseases in under-resourced regions (2–4). Consequently, the integration of climate change as a risk factor in educating health scientists was narrow and tentative. Increasing knowledge about the ways in which climate change impacts population health is now considered one of the major strategies for expanding public understanding of the challenges, and for encouraging political action beyond the rhetoric (5).

In response to the increasing understanding of the widespread adverse impacts projected to occur if the trend of greenhouse gas emissions leading to abrupt climate change is not reversed, the University of California established the Global Climate Leadership Council in 2014 with the goal of providing advice on strategies for achieving carbon neutrality by 2025. The UC GCLC was also charged with providing guidance on integrating carbon neutrality and sustainability goals into teaching, research and public service mission of the university. Specifically, the health sciences and services, faculty and student engagement were three of nine key areas of contribution for the council's organizational structure and function.

In this context, our initiatives focus on strategies to formally integrate the understanding of climate change impacts and sustainability broadly into the educational curricula across the health sciences and into healthcare practices. The first workshop was convened in Winter Quarter of 2020 with 20 faculty members in the Susan and Henry Samueli College of Health Sciences to integrate health impacts of climate change, including solutions, sustainability, adaptation, and resilience of the health sector into their existing courses. The College offers undergraduate (Bachelor of Science) degrees in Nursing Science, Pharmaceutical Sciences, Public Health Policy, and Public Health Science; graduate (Master of Science and Doctor of Philosophy) degrees in Nursing Science, Public Health (Global Health and Disease Prevention), Pharmaceutical Sciences, Epidemiology, Environmental Health, and in the Biomedical Sciences in collaboration with the School of Biological Sciences. The College also offers professional degrees in Medicine (Doctor of Medicine), Public Health (Master of Public Health), Nursing Science, and Pharmacy. Each participating faculty member receive $1,100 for transforming their course over the period of 1 year. The second workshop convened a year after the first one with presentations from each faculty member on how the transformed their courses, to discuss specific difficulties and to share best practices. A pre-workshop survey of participants was conducted with a 10-item questionnaire before the first workshop, including questions on their current teaching activities and their expectations for curriculum integrations regarding climate change. For example, to make recommendations about appropriate strategy for engaging students on the topic climate change in each course (Table 1), we asked participants to respond to the following question:

**Table 1:** Courses taught by workshop participants.

**Full course title**	**Undergraduate (UG) or graduate (G)**	**Enrollment size**	**Frequency quarters/year**
Public health statistics	G	23	1
Foundations of community health	UG	123	1
Research design	G	11	1
Public health practicum and upper division writing	UG	202	3
Cities: focal point for sustainability problems and solutions	UG	23	1
Natural disasters	UG	152	1
Environmental geology	UG	70	1
Compassionate care for underserved populations	G	20	1
Health communication R	UG	80	1
Health communication	G	17	1
Risk communication	G	10	0.5
Communities justice and health	G	36	1
Obesity epidemic	G	4	1
Health behavior change theory	UG	30	1
Introduction to community health sciences	G	30	1
Introduction to global health	UG	150	2
Global health ethics	UG	150	2
Cancer epidemiology	UG/G hybrid	50	1
Epidemiology in global health	G	15	1
Introduction to epidemiology	UG	163	1
Climate change and global health	UG	New	1
Theory of data analysis	G	11	1
Community-based healthcare	U	56	1
Speaking about science	UG	30	3
Research methods and applications in health care	UG	50	1
Advanced GIS and spatial epidemiology	G	20	1
Infectious disease dynamics	UG	New	1
Population dynamics in ecology, epidemiology, and medicine	UG	25	1
Computational modeling of diseases	G	New	1
Introduction to environmental health science	UG	70	1
Environmental health sciences	G	29	1

**“*In what format do you expect to integrate climate change information in your*** ***course(s)? Select all that apply:”***

a. *The entire course is about climate and health*
b. *A lecture on climate and health*
c. *Introduction or discussion of a case study or example of the impacts of climate on health*
d. *Assignment of articles for the students to read or videos to watch on climate and health*
e. *Guest lecture on climate and health*
f. *Other:______________________________*

## Models of curriculum integration

**Figure 1:**
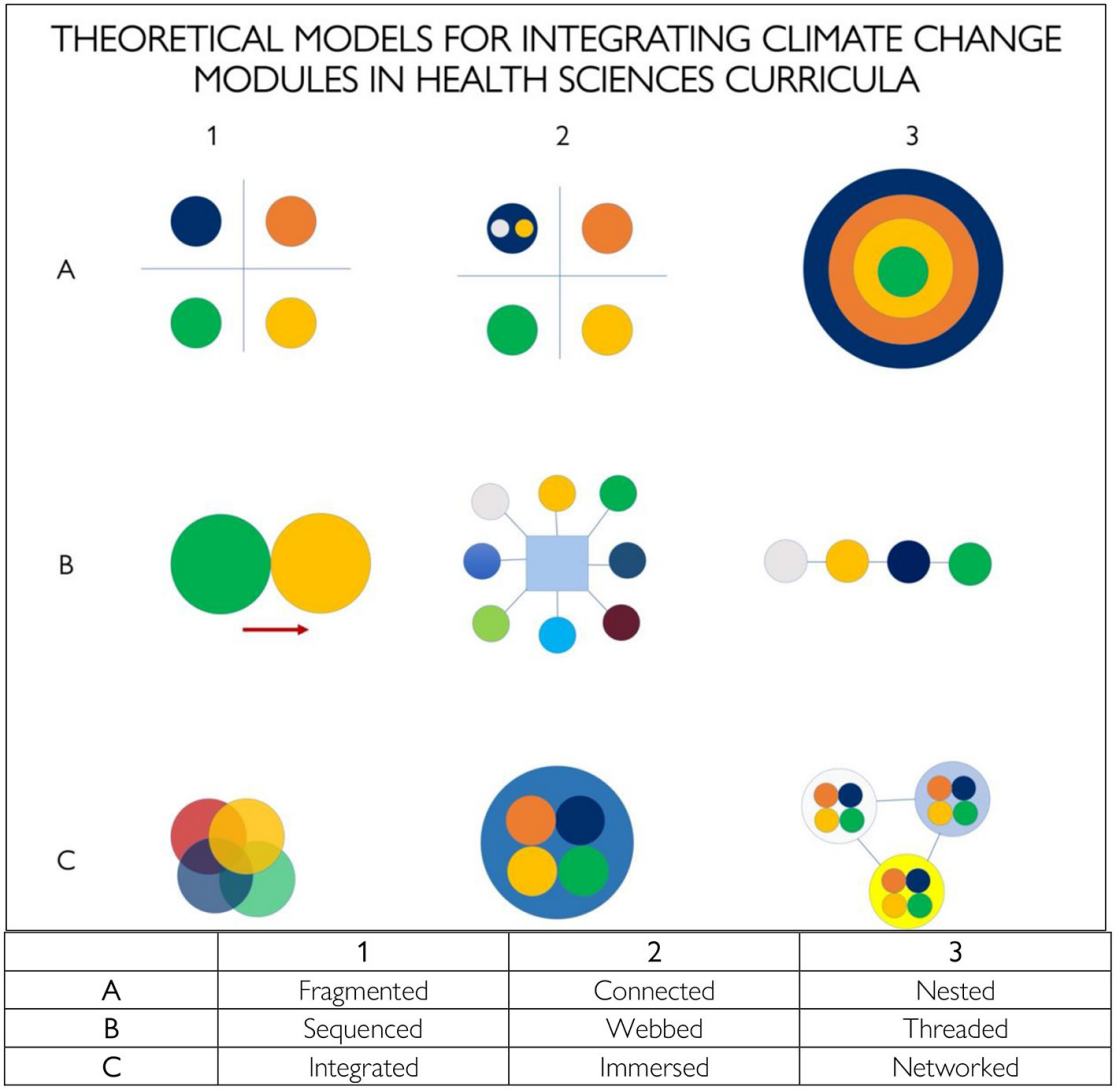
Diagrams of curriculum integration models suitable for including climate change in curricula health sciences curricula (7). A1, A2, and A3 models are strategies for courses within a single discipline or professional training; B1, B2, B3, and C1 are ideal for interdisciplinary courses that bring students from various schools together for interprofessional education; C2 and C3 are ideal for continuing professional development training.

The development of theoretical frameworks about “Curriculum Integration” (CI) is a very well-established subject of research in the academic and professional discipline of education. An integrated curriculum is typically designed to implement learning that is synthesized across two or more traditional disciplines and across a variety of experiences which reinforce the learning objectives (6). The theoretical models of CI include nine strategies which may serve different purposes for developing interdisciplinary curricula (Figure 1). For example, the traditional fragmented curricula that align with different professional training in health sciences have the advantage of presenting clear and distinct perspectives of the respective disciplines but is unsuitable for presenting climate change information because connections, for example, between the physical scientific basis of climate change are not well-connected to aspects of vulnerability, adaptation, and mitigation with respect to the health impacts. Therefore, the transfer of learning may be ineffective. Alternately, the connected and nested curriculum models may offer the opportunity to connect key concepts thereby generating assimilation of ideas within a discipline. Climate change-related modules and topics such as natural disasters, emergency preparedness, infectious disease outbreaks, air quality, heat stress, and water resources into existing courses can enhance the learning experience. A sequenced curriculum allows the introduction of a broad topic such as climate change followed by a more advanced presentation of specific topics related health outcomes and the role of healthcare providers and the healthcare system in responding to emerging threats. Climate change is also a suitable topic for interprofessional education where specific courses are designed for enrollment of students from various disciplines as exemplified by the webbed, immersed, integrated, and networked models (8). Problem-solving case studies and role plays are particularly suited for such interprofessional courses whereby trainees from different disciplines can debate and share various perspectives and methods to enrich the collective learning experience.

Thirty-one courses were presented for transformation with climate change information, among which there were 12 graduate-level courses, 19 graduate-level courses, including a course which simultaneously enrolls undergraduate and graduate students. Together, these courses enroll about 2,400 students annually. Among the courses considered for integrating climate change into the health sciences curricula, three were being developed including one specifically about climate change and global health. Enrollment data were not available for these three. The inclusion of datasets related to climate change was one of the most cited strategies for introducing the topic into quantitative courses such as public health statistics, theory of data analysis, and epidemiology courses. In this regard, the U.S. Environmental Protection Agency maintains a data-rich resource on climate change for educators and students (9). Modular case studies for reviewing, problem solving, and role play were typically considered for general health science courses such as risk communication, introduction to global health, and natural disasters. In this regard, the National Oceanic and Atmospheric Administration (NOAA) maintains an archive of teaching resources entitled “Teaching Climate” (10). The National Aeronautics and Space Administration (NASA) also maintains a resource website for educators (11). For physical science-oriented courses such as environmental geology, and natural disasters, the U.S. Geological Survey maintains a web archive for educational resources (12).

## Transforming “introduction to global health”

**Figure 2:**
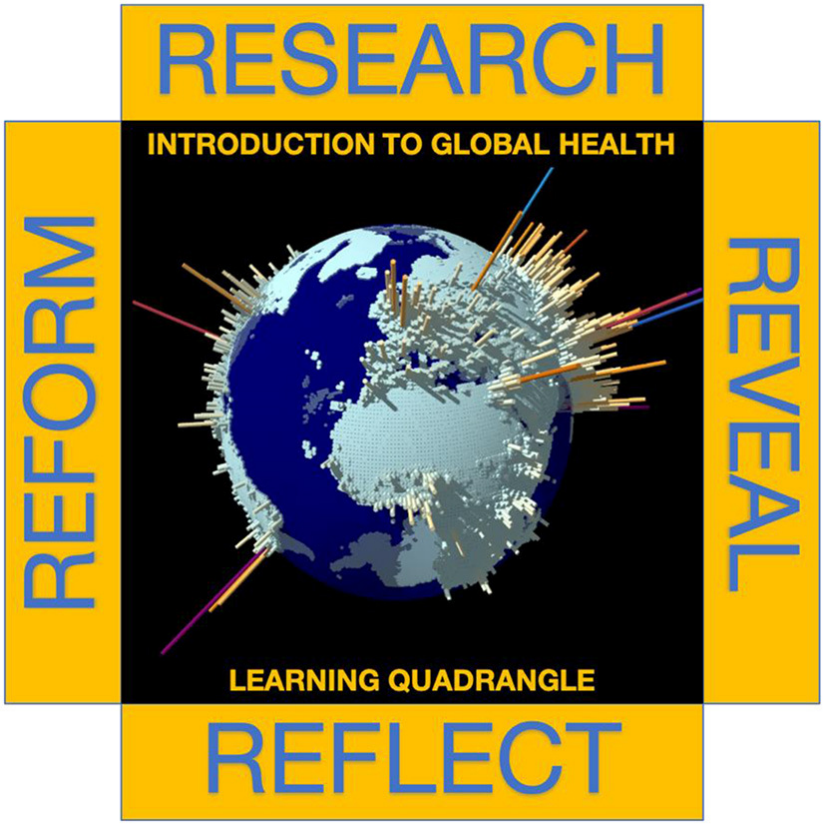
The *Learning Quadrangle* adopted in *Introduction to Global Health* uses a modular assignment structure to integrate special topics into the curriculum. Diagram of Earth with population density spikes by Anders Sandberg - https://www.flickr.com/photos/arenamontanus/375127836.

In 2015, the systemwide University of California Global Health Institute (UCGHI) began investing in the development of fully online asynchronous courses available for enrollment by students on all campuses (13). Funds for developing the courses were provided through a competitive request for proposals issued by the Innovative Learning Technology Initiative (ILTI; now known as UC-Online) (14). To promote interdisciplinary collaboration, curriculum integration, and cross-campus engagement, each proposal from UCGHI was expected to be developed and, if funded, implemented by faculty members from two or more UC campuses. “Introduction to Global Health” is among the earliest courses to be implemented. The course was co-developed by the author representing the biomedical perspective and Dr. Tom Csordas, the Founding Director of the Global Health program and Director of the Global Health Institute at UC San Diego, representing anthropological perspective. In addition, Dr. Laura Rosenzweig, staff at UCOP assisted as a professional course designer in production and implementation of the course. The adoption of a “Learning Quadrangle” framework facilitated course design in a modular format that aligned specific topics with weekly assignments in five sections *Inspiration* including readings and video recorded introduction of students to each topic (Figure 2). The inspiration section is followed by *Research* (guided and independent) whereby students are expected to read assigned documents and independently to find information including research and news articles, videos, and images relevant to the topic. This section reinforced the objective to have each student explore the topic on their own. The next section, *Reveal*, requires each student to post their findings in a community Asset Library and to construct concept maps using provided prompts. The next section, *Reflect*, invites students to review the concept maps of other students and to identify gaps in their own understanding or to specific their own unique perspective and conceptual understanding. The final section of the Learning Quadrangle, *Reform*, poses a challenging problem to be solved based on the student's understanding of the fundamental knowledge of the causative agents, current policies, and limitations in the realm of funding, political, or other socioeconomic difficulties. This sequential modular structure facilitated the review of an existing topic on *Global Health Impacts of Natural and Anthropogenic Disasters*. In this context, climate change is framed as an anthropogenic disaster because of the strong evidence linking human activities to greenhouse gas emissions, and consequently the increase in average global temperatures which forces changes in climactic conditions worldwide with projections of damage to quality of life and healthful conditions (15, 16). The case study of a climate-sensitive disease, Valley Fever (coccidioidomycosis) is used here as an example of curriculum integration because it covers climate science, epidemiological data, health impacts, vulnerable populations, health communication and adaptation strategies, and an argument for investing in mitigation strategies.

## Case study on valley fever

For more than a decade the incidence of coccidioidomycosis has increased steadily in the southwest United States where it is considered endemic. The disease develops in some individuals who are exposed through breathing the spores of the causative pathogen *Coccidioides imitis* present in soils. Dust from soils harboring *C. imitis* may be distributed widely under certain wind conditions, thereby creating vulnerability for populations over a wide geographic range. The ecological niche for *C. imitis* is defined by arid, desert zones where spores are found in lower elevations, >4 inches under sandy soil. Droughts, earthquakes, and building construction are all regarded as factors that increase vulnerability. The availability of epidemiologic data, climatic data, and social determinants of population vulnerability to coccidioidomycosis in response to climate change in California renders this case study suitable for the integration in to *Learning Quadrangle* teaching platform (17, 18). The *Inspiration* section of the module highlights the plight of patients who suffer from coccidioidomycosis, including a presentation on vulnerable domestic and agricultural animals. In the *Research* section students are guided to read peer-reviewed articles on the link between climate change and the expanding geographic range of *C. imitis*. Students may also bring articles that they discover about coccidioidomycosis outside California, including other states in the southwest region of the U.S., Central America and South America. In the *Reveal* section, students are expected to contribute concept maps to the Asset Library using the *Padlet* software linked to the *Canvas* learning platform. In the *Reflect* section, students compare and contrast their concept map on coccidioidomycosis, specific populations, climate data, occupational hazards, socioeconomic data, soil pH, soil composition, and weather patterns. In the final *Reform* section, students are given a prompt for which they respond by writing an essay, for example, on how to create a public information campaign for disease awareness and prevention; or a proposal to conduct research to fill gaps in knowledge. Students may also write about the inherent trade-offs in climate mitigation efforts and the potential impacts of statewide policies on climate mitigation on population health (19, 20).

## Conclusion

The application of theoretical models of curriculum integration can facilitate broad-spectrum integration of climate change education in health sciences curricular. The opportunity to adopt various models of curriculum integration including modification of existing course syllabi with new sub-topics, adding datasets for analysis, or presentation of case studies for guided research appeals to situations where addition of entirely new courses on climate change into a packed curriculum proves to be too difficult. Deep integration of climate change topics in courses which already address transboundary movement of people and environmental risk factors, for example in global health is facilitated by a learning quadrangle platform which supports independent and guided student engagement such that a wider range of topics are included in the climate change module beyond potential impacts on infectious disease epidemiology. Sub-topics within the climate change module include mental health impacts, damage to physical infrastructures which support water quality, waste management, energy supply, air conditioning, sea-level rise, food supplies, and inequity issues in access to health care. The models of curriculum integration presented in these workshops are applicable to in-person, hybrid, and online modes of course delivery, although the impacts of these modes on long-term impacts on the retention of climate change knowledge among students will require longer-term monitoring than review after a year of implementation. Institutionalization of workshops to integrate urgent topics into the curricula of health sciences professions can also serve topics beyond climate change, for example, the integration of diversity, equity, and inclusion into educational activities in the health sciences is becoming common. Incentives for faculty engagement and training-of-trainers workshops may be necessary to sustain wholescale transformation of saturated curricula in interprofessional education.

## Data availability statement

The original contributions presented in the study are included in the article/supplementary material, further inquiries can be directed to the corresponding author/s.

## Author contributions

OO conceived and executed the project and wrote the manuscript.

## Funding

Funds from the UCOP Climate Initiative, UC Online, and UCGHI supported for this work.

## Conflict of interest

The author declares that the research was conducted in the absence of any commercial or financial relationships that could be construed as a potential conflict of interest.

The reviewer KT declared a shared parent affiliation with the author to the handling editor at time of review.

## Publisher's note

All claims expressed in this article are solely those of the authors and do not necessarily represent those of their affiliated organizations, or those of the publisher, the editors and the reviewers. Any product that may be evaluated in this article, or claim that may be made by its manufacturer, is not guaranteed or endorsed by the publisher.
